# Negative experiences, social exclusion and unwanted attention on social media: exploring the association with adolescent alcohol use

**DOI:** 10.1186/s12889-022-14679-4

**Published:** 2022-12-16

**Authors:** Priya Ranganath, Gunnhild Johnsen Hjetland, Turi Reiten Finserås, Geir Scott Brunborg, Morten Hesse, Jens Christoffer Skogen

**Affiliations:** 1 Center for Alcohol and Drug Research, Aarhus University, Bartholins Allé 10, 8000 Aarhus, Denmark; 2 Department of Health Promotion, Norwegian Institute of Public Health, Bergen, Norway; 3 Centre for Evaluation of Public Health Measures, Norwegian Institute of Public Health, Oslo, Norway; 4 Department of Alcohol, Tobacco and Drugs, Norwegian Institute of Public Health, Oslo, Norway; 5 Department of Clinical Neuroscience, Karolinska Institutet, Stockholm, Sweden; 6 Alcohol and Drug Research Western Norway, Stavanger University Hospital, Stavanger, Norway

**Keywords:** Drinking, Online, Isolation, Adolescent, Binge, Coping

## Abstract

**Background:**

Adolescents’ presence on Social Media (SoMe) facilitates peer connections making them susceptible to peer-influences and approval. Negative experiences on SoMe can affect adolescent stress and wellbeing, impelling their use of alcohol. This paper provides a novel understanding of the relationship between negative experiences on SoMe and key indicators of alcohol use in adolescents.

**Methods:**

Data was collected from upper secondary school students (*n* = 3528, ages 16–19, 45% boys) in Bergen (Norway) using a web-based questionnaire during school-hours in 2020 and 2021. Dependent variables were alcohol consumption, binge drinking and scoring ≥ 2 points on the CRAFFT instrument screening for substance use problems in adolescents. Independent variables were two scales indicating “negative acts and exclusion” and “unwanted attention from others”. Covariates included age, gender, country of birth and subjective socioeconomic status. Results are presented as relative risk ratios (RRR), odds ratios (OR) and incidence rate ratios (IRR) with 95% confidence intervals.

**Results:**

Using multinomial logistic regression models, “negative acts and exclusion” and “unwanted attention” were positively associated with trying alcohol (OR: 1.50 (95% CI 1.28–1.76) and 1.86 (95% CI 1.66–2.09) respectively, both *p*** ≤ **0.001), with frequency and amount of alcohol consumed. Findings from logistic regression models indicated that “negative acts and exclusion” and “unwanted attention” were positively associated with i. CRAFFT-caseness (OR: 2.13 and 1.86) and ii. frequent binge drinking (OR: 1.55 and 1.89).

**Conclusion:**

Both exclusion and unwanted attention on SoMe were associated with indicators of problematic drinking, including frequency, quantity, and consequences related to alcohol.

## Background

Adolescent alcohol use is one of the most important risk factors for reduced health among young people [1] and it is associated with a greater risk of adversity and social exclusion later in life [2]. The period of adolescence is marked with trials and adjustments where adolescents explore social connections and may participate in high-risk behaviour (e.g. substance use, unsafe sexual acts; [3]). Adolescents may satisfy their need to remain socially connected by living in a virtual world where they meet and maintain relationships with their peers through the internet, especially on Social Media (henceforth, SoMe; [4, 5]). In Western Europe and Nordic countries, 95% of adolescents use social media each week and at least 2–3 h on a school day [6]. In today’s society, SoMe tends to be an arena for social interaction (where negative interactions may have a strong adverse effect on well-being). Through online connections, adolescents seek to explore self-identities, peer relationships, acceptance, and approval [7], learning to optimise their online personalities to gather positive feedback [8]. Adolescents place high merit in social comparison, self-disclosure, and impression management, which make them highly sensitive to feedback and peer influence [4, 9–11]. It is now evident that interactions and relationships on SoMe provide adolescents with a sense of security and belonging, enabling them to express and satisfy enhanced risk-taking needs [7, 12]. However, there is still a debate concerning SoMe’s potential protective effects on adolescents’ risky behaviour [4, 13].

### Social media and alcohol use in adolescents

Adolescent drinking continues to be a public health concern, and SoMe has emerged as a powerful medium to communicate with adolescents about their health choices; including their displays of drinking online or drinking practices while one is offline [14, 15]. In particular, SoMe may also function as an arena that encourages cultures of intoxication [15], modeling drinking behaviour to adolescents. Some argue that the rapid increase in the use of SoMe has likely displaced engagement in harmful behaviours such as excess alcohol consumption [16, 17] while others suggest that SoMe provides a new context that increases adolescents’ susceptibility to risky behaviour. Recent literature suggests that the desire for social connectedness is often a driver of recreational drug use [18] with high peer connectedness presenting a risk for problematic use of substances [19]. Research amongst adolescents has indicated that adolescents who use SoMe [20] and view alcohol-related content [21] have a greater likelihood of using more alcohol or drinking heavily compared to those who does not use SoMe [22]. This study found that the use of SoMe was also associated with past month binge drinking in the same cohort [22]. Furthermore, a recent longitudinal study of Norwegian adolescents found a positive association between time spent on SoMe and a subsequent increase in alcohol use over time [23]. Westgate and Holliday [24] discovered that alcohol-related content was linked to higher rates of alcohol use, craving, alcohol-related problems, and clinical alcohol use disorders. Taken together, SoMe use influences both dimensions of adolescent drinking – consumption of alcohol as well as drinking at hazardous/problematic levels. It is vital to study both dimensions as initiating and continuing alcohol use in adolescence is frequently associated with the development of unhealthy drinking patterns as well as subsequent alcohol use disorders in adulthood [25–27].

A recent meta-analysis on alcohol-related SoMe use and consequent alcohol consumption in adolescents reiterated that greater alcohol-related engagement (content and posts) was indeed correlated with both greater self-reported drinking and alcohol-related problems [28]. Similarly, other recent studies have illustrated that alcohol-related posts heightened depression, anxiety, and the potential for risky drinking [12, 29, 30]. Others state that alcohol-related exposure might cause lenient alcohol-related cognition that is frequently interpreted as positive peer endorsement of alcohol-behaviour [31]; and, that online displays of alcohol use and related content have likely normalised its use in adolescents [32]. It has been suggested that this normalization might create “intoxigenic digital spaces”, where alcohol consumption becomes associated with something cool or fun [33, 34].

However, beyond assisting with modeling drinking behaviour, the internet is also an important social arena for many adolescents, and positive or negative experiences may have a deep impact on their well-being and behaviour. It has thus been suggested that increased alcohol consumption is linked to negative online experiences such as rejection and isolation [33, 34] where alcohol consumption becomes a coping strategy [35]. More research is required on adolescent SoMe experiences, settings, and contexts, and its relationship to their alcohol use. For instance, it is important to determine the degree to which adolescents are having negative experiences on SoMe, and whether these are associated with alcohol use. Therefore, the focus of this paper is to study the potential associations between these negative experiences on SoMe and drinking behaviours in adolescents.

### Negative experiences on social media

#### Negative acts and exclusion

While the use of SoMe presents new socialising experiences that help develop identity and belonging, exclusion on SoMe has been shown to affect one’s sense of belonging, meaningful existence, self-esteem, and sense of control [36]. A growing body of literature has explored the effects of social exclusion on SoMe [37–39], oftentimes referred to as *cyber ostracism* [40] which is defined as the feeling of being ignored or excluded on online platforms. Humans are highly sensitive to social exclusion, and being unfriended, not receiving a reply after one’s message is denoted as “read”, not being tagged in the social post of another, or not receiving the usual amount of *Likes,* could be interpreted as ostracism. These have similar consequences to being excluded in other social contexts [37, 41–43]. Social rejection has been shown to increase the likelihood of drinking alcohol among young adults who drank regularly [44]. Among people with an existing alcohol problem, exclusion may perpetuate unhealthy drinking habits [39]. Drinkers reported that alcohol helps them face stressful situations and provides a way of coping with negative experiences of social exclusion, both online and offline [44, 45]. To further illustrate, Laws et.al [44] state that due to dysregulating aspects of social rejection including increased arousal and negative affect, individuals may turn to alcohol use as a means of stress reduction while the same social rejection might also prompt individuals to seek greater social belonging and euphoria, which is commonly found in social drinking. Similarly, Pichel et.al [8] ascertained that alcohol use became a coping strategy for many adolescents who use SoMe for social exploration and risky online presentation—both for the instigator and receiver of negative experiences.

#### Unwanted attention from others

Another potentially stressful aspect of negotiating peer relations on SoMe is the unwanted attention from family, online friends, or strangers. Unwanted attention can be used to describe a range of intrusive behaviours that could constitute a pattern of stalking or isolated experiences that one perceives as unacceptable [46]. It has been measured in a multitude of ways in recent literature but conceptually, it denotes problematic and prejudiced themes around one’s identity, sexuality and gender [47]. For instance, using findings from the Third Youth Internet Safety Survey, Mitchell and colleagues [48] described unwanted online experiences as unwanted sexual solicitations, harassment, and unwanted exposure to sexual material. While unwanted sexual solicitations were reported mainly by adolescent females between the ages of 16 and 17, harassment was more frequently observed amongst adolescent females aged 13–15. Unwanted exposure to sexual materials was reported by adolescents of all ages [48, 49]. More recently, Henry and Powell [47] described cyber sexual harassment as the experience of receiving unwanted sexual messages/photos, unwanted requests (or pressure) to send sexual messages/photos, having sexual messages/photos shared without consent, and unwanted solicitation to do something sexual.

Reactions to unwanted attention could vary between people. Some might feel put off and inconvenienced by unwanted solicitation and attention, while others may feel violated as their security and psychological well-being are threatened. Adolescents who were victims of internet harassment and online sexual solicitations reported psychosocial problems including alcohol and marijuana use, at an elevated level compared to those who experienced little or none [50]. In a study on cyber sexual harassment amongst 15–19-year-old girls (*n* = 159), participants who were harassed reported greater odds of reporting past 30-day alcohol use, past 30-day binge drinking, and lifetime drug use [51].

Although repeated harassment or cyberbullying, sexting, and digital dating abuse have captured the attention of researchers and the public alike, the prevalence and consequences of negative experiences and unwanted attention on SoMe amongst adolescents as well as its impact on alcohol use, is less documented. In addition, to our knowledge, no studies to date have examined a potential dose–response effect between negative experiences on social media and alcohol consumption, where more exposure (negative experiences on social media) presents a greater impact (more alcohol use). Due to the dearth of evidence and studies in this area of alcohol-use research, the objective of this paper is to provide a novel understanding of the relationship between negative experiences on SoMe and key indicators of alcohol use in adolescents. Using data from a study of upper secondary school students (*n* = 3528) in Bergen (Norway), this study aims to examine the role of two specific negative experiences on SoMe i.e. i. “negative acts and exclusion”, and ii. “unwanted attention from others” on alcohol use and binge drinking in adolescents.

## Methods

### Participants

Participants were recruited from Upper secondary schools in Bergen, Norway. Upper secondary schools in Norway comprise grades 11 to 13 usually consist of students who are 15–16, 16–17, and 18–19 years respectively. Most students start in the autumn of the year when they turn 16 and thestudents had to be 16 years or more to participate. There was no upper limit to the age range however, the group of older students (aged 20 and 21) constituted only 4% of the total sample. Data collection was done over two time-points in 2020 and 2021, and the participation rate was 53% and 35.4%, respectively (of those initially invited to participate) with a total of 3,528 (98%) eligible for the present analyses. The median age of the participants was 17 and 45% were boys.

### Procedure

This study used data from the "LifeOnSoMe" study of upper secondary school students in the city of Bergen, Norway [10, 52]. Data were collected using a web-based questionnaire during school hours. The students received a survey-specific web address which led them to written information about the study and they could also consent to participate. The study was approved by the regional ethics committee and is in agreement with the General Data Protection Regulation (See “Ethics” below for more details).

### Dependent variables

#### Measures of alcohol consumption

All participants could indicate whether they had tried alcohol or not (yes or no). Those responding *yes* were also asked how frequently they usually consume alcohol in a 14 days period (ranging from “less than once” to “5 times or more”), and how many units they usually consume while drinking (ranging “from 1–2 units” to “10 + units”).

#### Binge drinking

The participants who had tried alcohol were also asked how often they had consumed so much alcohol that they were clearly intoxicated (“drunk”), ranging from “Never” to “More than 20 times” in their lifetime (see Table 2 for more information on the range on this variable). The variable “Frequent binge drinking” was defined as reporting “more than 20 times”, as this was the highest possible category and relatively common (14%) in the present study.

#### Alcohol-related problems: CRAFFT

The CRAFFT is a screening instrument for alcohol and drug-related problems in children, adolescents, and young adults [8]. The instrument’s name is a mnemonic of the first letters of keywords in the six items (see Table 1) that ask respondents about alcohol-related events in three contexts (when going by Car, to Relax, when Alone) and three negative consequences (Forget things while using alcohol, Friends telling you to cut down, and getting in Trouble while using alcohol, see Table 1). For the participants having tried alcohol, we presented them with a modified version of the CRAFFT questionnaire which specifically gauges potential alcohol-related problems. The participants indicated their answers as “yes” (1) or “no” (0), giving a total score between 0 and 6, where a higher score indicates more problematic alcohol use.

**Table 1:** The CRAFFT interview questions

	Description	Items #
**C**	Have you ever ridden in a **C**AR driven by someone (including yourself) who was “high” or had been using alcohol?	Variable 1
**R**	Do you ever use alcohol to **R**ELAX, feel better about yourself, or fit in?	Variable 2
**A**	Do you ever use alcohol while you are by yourself, or **A**LONE?	Variable 3
**F**	Do you ever **F**ORGET things you did while using alcohol?	Variable 4
**F**	Do your **F**AMILY or FRIENDS ever tell you that you should cut down on your drinking?	Variable 5
**T**	Have you ever gotten into **T**ROUBLE while you were using alcohol?	Variable 6

Moderate internal consistency reliability for CRAFFT (Cronbach’s alpha = 0.67) was reported for adolescents aged 17–19 in Sweden and Norway [53, 54]. In our sample the composite reliability was 0.78, indicating a moderate-to-high internal consistency. Participants with a score of 2 or more were categorized as having alcohol and/or drug-related problems, termed CRAFFT-caseness [54].

### Independent variables

#### Negative experiences on social media

Respondents rated eight statements regarding negative experiences on social media, on a five-point Likert scale ranging from “Never” to “Very often”. The statements are derived from analyses of focus group interviews of adolescents regarding social media use and mental health and well-being [52]. Examples of statements are “I receive unwanted nude pictures/sexualised content” and “I feel excluded from groups/chats” (For the complete list, see reference [55]).

Based on a previous study investigating the dimensionality of these statements [55], variables 1, 3, and 4 were combined as a composite measure of “Unwanted attention from others” (composite reliability 0.89), and the remaining five variables were combined as a composite measure of “Negative acts and exclusion” (composite reliability 0.92). Lastly, we also estimated the number of endorsed items (i.e. more than “never”) ranging from 0 (23.1%) to 8 (7.4%). This count variable was named “Number of negative experiences”.

#### Age, gender, and country of birth

Age and gender were registered by self-report. Gender included a non-binary option, but only 37 participants ticked this option. Due to the small number, they were excluded from further analyses in the present study. The participants could also indicate where they were born, and in the present study, we differentiate between being born in Norway (90%) and being in another country.

#### Subjective socioeconomic status

Subjective socioeconomic status (S-SES) was gauged by the question “How well off do you consider your own family to be compared to others?”, ranging from 0 (“Very poor”) to 10 (“Very well off”) [56].

### Statistical analysis

**Fig. 1:**
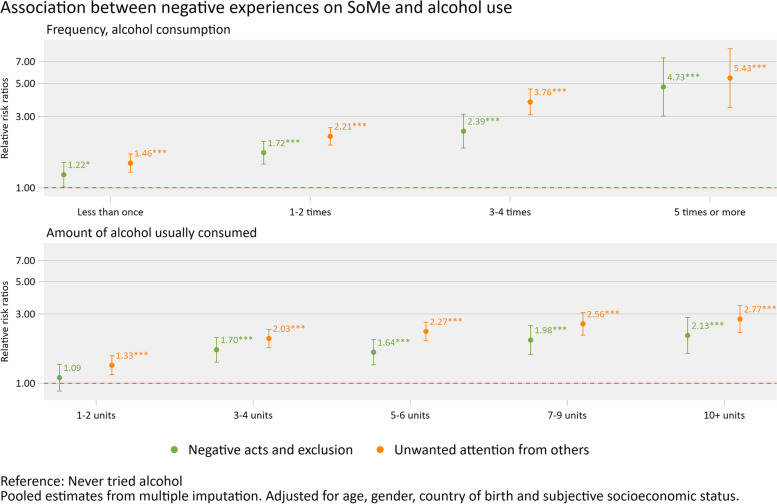
Association between negative experiences on SoMe and alcohol use

**Fig. 2:**
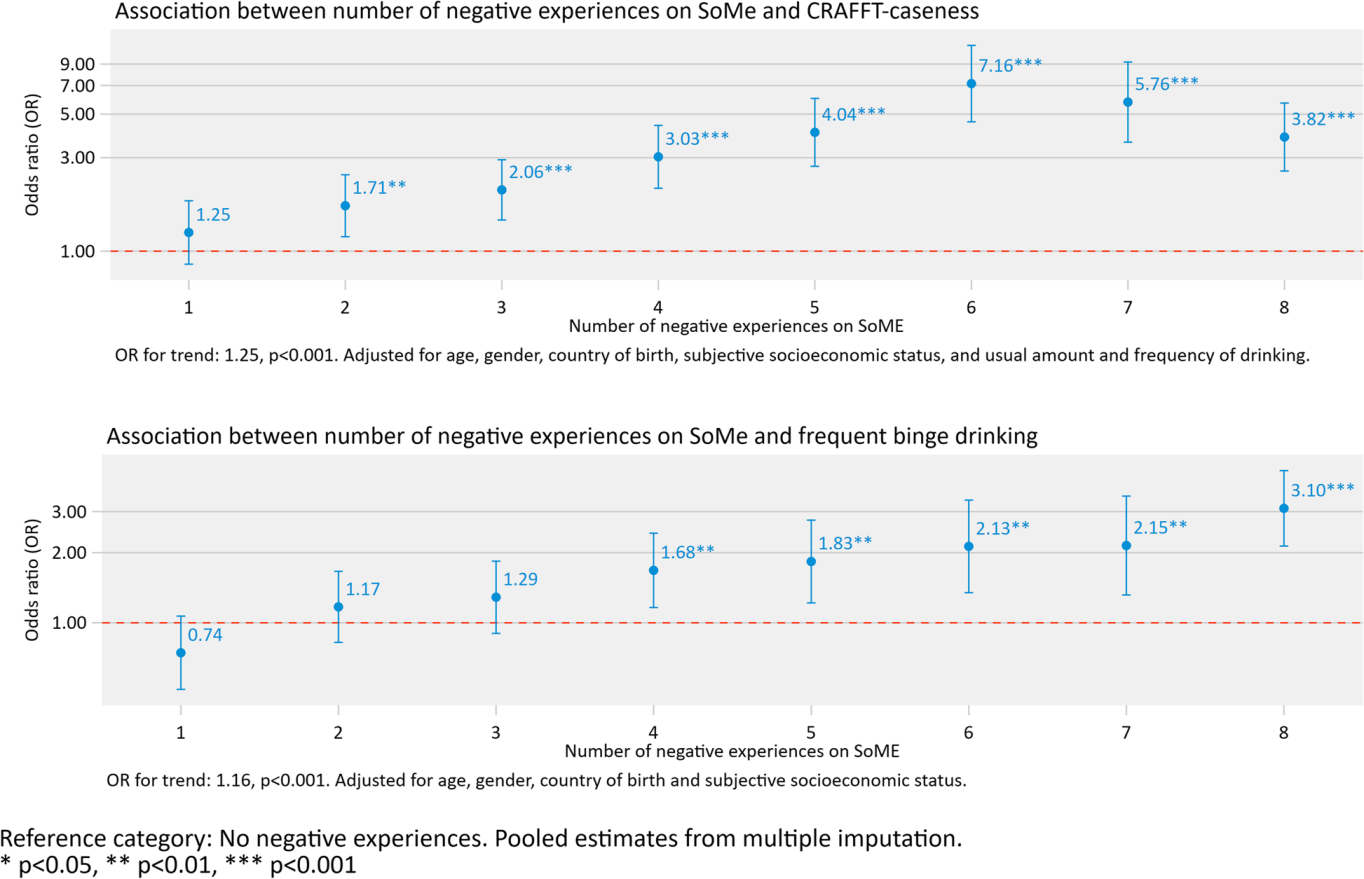
Association between number of negative experiences on SoMe and CRAFFT-caseness and frequent binge drinking

First, results from descriptive analyses of the included variables are presented in Table 2, using mean and standard deviation for continuous data; median and interquartile range for count data, and, frequency and proportion for categorical data. Second, the association between negative experiences and having tried alcohol adjusted for age, gender, country of birth and S-SES was estimated using separate logistic regression models for the variables “negative acts and exclusion” and “unwanted attention from others”. Third, the association between “negative acts and exclusion” and “unwanted attention from others” and frequency of alcohol use and amount of usual consumption was estimated using multinomial logistic regression models adjusted for age, gender, country of birth, and S-SES (Fig. 1). The results are presented as relative risk ratios (RRR) with 95% confidence intervals (95% CI) using “never tried alcohol” as the reference category. Fourth, the association between negative experiences and CRAFFT-caseness when adjusted for age, gender, country of birth, and usual amount and frequency of drinking was estimated using logistic regression models across the variables “negative acts and exclusion”, “unwanted attention from others” and “number of negative experiences” (presented as odds ratios with 95% CI). Fifth, the association between negative experiences and frequent binge drinking when adjusted for age, gender, country of birth, and S-SES was estimated using logistic regression models across the variables “negative acts and exclusion”, “unwanted attention from others” and “number of negative experiences” (presented as odds ratios with 95% CI). For CRAFFT-caseness and frequent binge drinking, the participants reporting never having tried alcohol were included in the group below the cut-off for CRAFFT-caseness and frequent binge drinking (i.e. scored “0”) with the above reference category. The results across the number of bad experiences are presented in Fig. 2. Fifth, the association between negative experiences on social media and score on CRAFFT when adjusted for age, gender, country of birth, S-SES, and usual amount and frequency of drinking was estimated using negative binomial regression models across the variables “negative acts and exclusion” and “unwanted attention from others”, and “number of negative experiences”. For those reporting never having tried alcohol, the CRAFFT score was set to “0”. The results from the negative binomial regression models are presented as incidence rate ratios (IRR) with 95% CI in Table 3.

**Table 2:** Summary statistics of all variables in the study

Characteristic	% or M(SD)
Age	
16	656 (19%)
17	1,672 (47%)
18	963 (27%)
19 +	237 (6.7%)
Gender	
Boys	1,573 (45%)
Girls	1,955 (55%)
Country of birth	
Norway	3,186 (90%)
Other	339 (9.6%)
Missing	3
Subjective socioeconomic status	7.15 (1.8)
Missing	47
Number of bad experiences on SoMe	2.00 (1.00, 4.00)^a^
Negative acts and exclusion, SoMe	1.41 (0.57)^a^
Missing	226
Unwanted attention from others, SoMe	1.86 (0.89)
Missing	229
Ever tried alcohol	2,563 (73%)
How often do you drink alcohol?	
Less than once	987 (28%)
1–2 times	1,346 (38%)
3–4 times	182 (5.2%)
5 times or more	23 (0.7%)
Never tried alcohol	965 (28%)
Missing	25
How much do you drink?	
1–2 units	663 (20%)
3–4 units	623 (19%)
5–6 units	614 (18%)
7–9 units	313 (9.3%)
10 + units	181 (5.4%)
Never tried alcohol	965 (29%)
Missing	169
Binge drinking	
Never	622 (18%)
Yes, once	247 (7.0%)
2–3 times	415 (12%)
4–10 times	415 (12%)
11–20 times	347 (9.9%)
> 20 times	503 (14%)
Never tried alcohol	965 (27%)
Missing	14
Frequent binge drinking	
No	2,046 (58%)
Yes	503 (14%)
Never tried alcohol	965 (27%)
Missing	14
Score on CRAFFT	0.86 (1.21)
Missing	4
Case-level CRAFFT	
No (< 2)	1,700 (48%)
Yes (2 +)	859 (24%)
Never tried alcohol	965 (27%)
Missing	4

^a^Median (IQR)

*N* = 3,528

**Table 3:** Association between negative experiences and score on CRAFFT

Characteristic	IRR^a^	95% CI^a^	*p*-value
Negative acts and exclusion, SoMe	1.31	1.24, 1.38	< 0.001
Unwanted attention from others, SoMe	1.28	1.23, 1.33	< 0.001
Number of bad experiences. SoMe			
0	—	—	
1	1.04	0.90, 1.21	0.6
2	1.28	1.11, 1.48	< 0.001
3	1.32	1.15, 1.52	< 0.001
4	1.54	1.34, 1.78	< 0.001
5	1.82	1.57, 2.11	< 0.001
6	1.97	1.68, 2.31	< 0.001
7	1.92	1.63, 2.25	< 0.001
8	1.83	1.59, 2.11	< 0.001

^a^*IRR* Incidence Rate Ratio, *CI* Confidence Interval

Adjusted for age, gender, country of birth, subjective socioeconomic status, and usual amount and frequency of drinking

Data handling and analysis were performed in Stata version 15 [57] and R using RStudio. To accommodate for missing values across different analytical models, multiple imputations using multivariate imputation by chained equations were employed and *n* = 20 imputed datasets were estimated.

## Results

### Descriptive statistics

Descriptive statistics are provided in Table 2 along with mean, median, and standard deviation presented where appropriate. Of all respondents (*n* = 3528), 73% (*n* = 2563) reported having ever consumed alcohol with 53.3% (*n* = 1368) of these having consumed “1–2 times”. In terms of the dependent variables, those who had consumed 5 to 10 units of alcohol consisted of 43.2% (*n* = 1108). Frequent binge drinkers, defined in this study as having been “drunk” 20 times or more, included 20% of respondents (*n* = 503). However, close to 22% of respondents had been drunk anywhere between 4 to 20 times. The mean CRAFFT score was 0.86 (standard deviation: 1.21) with 33.5% (*n* = 859) having a CRAFFT-caseness score of 2 or more.

While considering the independent variables, the median (IQR) for the number of bad experiences on SoMe was 2.00 (1.00–4.00).  The mean (SD) of the two dependent variables was 1.41 (0.57) for “negative acts and exclusion” and 1.86 (0.89) for “unwanted attention from others.

### Association between negative experiences on SoMe and alcohol use

Experiencing “negative acts and exclusion” and “unwanted attention from others” were adjusted for age, gender, country of birth, and S-SES and positively associated with having tried alcohol (OR: 1.50 (95%CI 1.28–1.76) and 1.86 (95%CI 1.66–2.09) respectively, both *p*-values < 0.001). Furthermore, results from multinomial logistic regression models adjusted for age, country of birth, gender, and S-SES indicated that both “negative acts and exclusion” and “unwanted attention from others” were positively associated with the frequency of consumption and the usual amount of alcohol consumed (See Fig. 1).

### Association between number of negative experiences on SoMe and a) CRAFFT-caseness and b) frequent binge drinking

Findings from logistic regression models adjusted for age, gender, country of birth, S-SES, and usual amount and frequency of drinking indicated that both “negative acts and exclusion” and “unwanted attention from others” were positively associated with CRAFFT-caseness (OR: 2.13 (95%CI 1.81–2.51) and 1.86 (95%CI 1.66–2.09), respectively, both *p*-values < 0.001). For frequent binge drinking, positive associations between “negative acts and exclusion” (OR: 1.55 (95%CI 1.34–1.81)) and “unwanted attention from others” (OR: 1.89 (95%CI 1.70–2.10)) were observed in logistic regression models adjusted for age, gender, country of birth and S-SES.

A higher number of bad experiences was in general positively associated with CRAFFT-caseness (OR for trend: 1.25, *p* < 0.001) and frequent binge drinking (OR for trend: 1.16, *p* < 0.001) (Fig. 2). The trends were fairly consistent across number of bad experiences, except for a dip in reporting seven or eight negative experiences on SoMe concerning CRAFFT-caseness.

### Association between negative experiences and scores on CRAFFT

Findings from negative binomial regression models adjusted for age, gender, country of birth, S-SES, and usual amount and frequency of drinking (with pooled estimates from multiple imputations) showed that both “negative acts and exclusion, SoMe” (incidence rate ratios (IRR): 1.31) and “unwanted attention from others” (IRR: 1.28) were positively associated with the score on CRAFFT (see Table 3). A higher number of bad experiences was positively associated with scores on CRAFFT (IRR for trend: 1.09 (95%CI 1.08 -1.11) *p* < 0.001) (Table 3). The trends were, however, not monotonic, but relatively consistent.

### Discussion

Adolescent alcohol use has declined in recent years. Still, in 2019 over half of Norwegian 15/16-year olds reported lifetime drinking, and nine percent reported drinking to intoxication in the last 30 days [58]. Studying the reasons why Norwegian adolescents engage in alcohol use is important for effective prevention of harmful consumption and related disorders. A growing number of publications have examined the correlation between alcohol-related engagement on SoMe and alcohol use, but few have explored the potential mechanism behind why some adolescent users of SoMe drink more and with greater frequency than others. In this paper, we postulate the contribution of their negative experiences on SoMe to their increased drinking.

Using data from an upper-secondary school-based study, we investigated the association between negative events on SoMe (namely i. negative acts and exclusion and ii. unwanted attention) and alcohol use amongst adolescents. After adjusting for age, gender, country of birth, and subjective socioeconomic status, we found that both social exclusion and unwanted attention on SoMe were associated with frequency and quantity of drinking (crude associations were similar to those presented across analytical models). Even after adjusting for age, gender, country of birth, subjective socioeconomic status as well as usual amount and frequency of drinking, both social exclusion and unwanted attention on SoMe was consistently associated with potential alcohol-related problems as measured by CRAFFT. Both these findings indicate a positive association between negative experiences and alcohol consumption in adolescents – whether it be indicators of alcohol consumed (as indicated by frequency and amount) or problematic drinking (as indicated by the CRAFFT scores). Therefore, further study of “negative acts and exclusion” and “unwanted attention from others” on SoMe might provide more perspective on the continuity of drinking as well as on its progression to problematic drinking in adolescents.

Our findings suggest the presence of a type of dose–response relationship [59, 60] where the dose model posits that more exposure to stimuli presents a greater impact. To our knowledge, there are no studies that have examined negative experiences on SoMe using a dose–response model; where the relative risk of binge drinking and negative outcomes of drinking increases with increasing negative experiences on SoMe. Our findings support past research suggesting that exposure to negative experiences on SoMe may be associated with detrimental consequences of adolescent drinking frequency and patterns [39, 50, 51].

SoMe was explicitly designed to connect individuals as well as impel positive feedback [5] via comments and *likes*, thus most adolescents mainly receive positive feedback that stimulates their sense of belonging and self-esteem. However, the potential impact of SoMe use may differ across adolescents and sub-groups. A recent study on adolescent SoMe use demonstrated that 10% experienced negative effects of passive social media use, while 44% felt neither better nor worse and 46% felt better [13]; indicating that the effects of SoMe use depend on individual and social factors.

We suggest two probable explanations for the differing impact across adolescents using SoMe – the effect of major life events and the influence of socioeconomic status. Adolescents in particular experience several life transitions, both positive and negative. They might constrain or turn to SoMe to disclose in order to regain normalcy and develop new identities. Processing harsh life transitions is a social adjustment and is bound to magnify the effect of any negative experiences online, which could lead to maladaptive behaviours (e.g., risky sex, and heavy alcohol use). The Major Life Events Taxonomy presented by Haimson et al. [61] lends support by illustrating that major life events or transitions (death of a loved one, childhood trauma, gender transition, truancy etc.) affect people’s behaviours, their use of technology and subsequent health outcomes. Secondly, in a study of Norwegian adolescents and negatives experiences on SoMe, Skogen and colleagues [55] discerned that the number of negative experiences reported on SoMe increased by 1.26 times for those with low socioeconomic status compared to those with high socioeconomic status, and by 1.10 times for those with medium socioeconomic status, even after adjusting for age, gender and amount of SoMe use. Future research on adolescent SoMe use should consider the cumulative effects (both negative and positive) of major life events and socio-economic status on negative experiences online.

### Strengths and limitations

The current study demonstrates various strengths. Firstly, this is the first study, to our knowledge, that has investigated the potential association between negative experiences on SoMe and alcohol consumption in adolescents and young adults. Secondly, data collection specifically focused on different aspects of SoMe use in adolescents. Finally, data collection was robust and recent with a large number of participants enabling a purposeful exploration into negative experiences on SoMe, the frequency of these experiences, and alcohol use outcomes in young adults.

Some important limitations of the present study need to be considered. First, there is a possibility that common methods variance would contribute to the correlation since both SoMe experiences and drinking behaviour were assessed using self-report. We cannot fully rule out reverse causality, in that adolescents who drink heavily may have negative interactions with others while drinking, which may lead to later negative or unwanted attention on SoMe, or that the posting of pictures or comments related to heavy drinking may contribute to some of the negative or unwanted attention. Also, while we directly assessed the diversity of negative experiences on SoMe, we did not assess how they were experienced or other variables that could affect how negatively they would affect the adolescents (e.g., negative comments from random strangers could potentially have less of an effect than negative comments from classmates). The current study has endeavored to include important controls. However, other confounders such as poor mental health, parental supervision, and sexual orientation, which might shed more light on the relationship between the dependent and independent variables, are beyond the scope of the study.

Furthermore, we did not assess the potential role of mental health and well-being in the relationship between negative experiences on social media and alcohol use. Social media use has been linked to adolescent mental ill-being (e.g., symptoms of anxiety and depression [62]. Negative experiences on social media may lead to lower well-being, in turn leading to increased alcohol use. Future longitudinal studies should assess the potential mediation of mental health and well-being on the relationship between negative experiences on social media and alcohol use.

### Implications and future research

A plethora of academic research has identified both negative short-term and long-term adverse effects of alcohol-related problems in adolescents, including school performance issues and truancy [63, 64]; social exclusion/isolation [65, 66]; peer and parental problems [67]; cognitive/developmental problems [68, 69]; mental health issues [65, 70]; increased risk for alcohol use disorders [71] and, poor occupational outcomes [72, 73]. Our findings indicate that one risk factor might be negative experiences on SoMe that potentially make adolescents more predisposed to follow peer-influencing and risky norms of alcohol use. Several studies have also shown that alcohol-related content on social media is associated with alcohol use [12, 24]. However, future research in the field of social media and substance use should not just focus on alcohol-related content and marketing but also on interactive experiences. Future studies could explore whether having negative experiences on social media is related to being exposed to alcohol-related content, or if alcohol-related content explains more of the variance in problematic alcohol consumption. More research is also needed to understand how this knowledge can be translated into preventative and intervention strategies in different settings, both online and offline.

From a clinical perspective, clinicians need to attune to adolescent interactions and experiences, both offline and on SoMe. Our findings reiterate that alcohol use amongst adolescents is a public health, as well as a clinical problem, where interactions and experiences on SoMe increasingly manifest as problematic consumption of alcohol. Studying the experiences of adolescents and their alcohol use on SoMe provides more nuanced information that can inform clinical practice. Finally, SoMe should be considered in intervention and prevention strategies targeting adolescents’ alcohol use. Clinicians must actively interact with adolescents on SoMe and provide access to clinical resources on drinking behaviour and associated concerns.

## Conclusion

This study has shown that negative experiences on SoMe are associated with hazardous alcohol use among adolescents. These results help clarify earlier research results on the association between adolescent SoMe use and heavy drinking. Future research should employ longitudinal design to establish causality in the relationship between negative experiences on SoMe and alcohol consumption.

## Data Availability

Explicit consent from the participants is required by the Norwegian Health research legislation and the Norwegian Ethics committees in order to transfer health research data outside of Norway. Ethics approval for this study was also was dependent on storing the research data on secure storage facilities located at the Norwegian Institute of Public Health, which prevents the authors from providing the data as supplementary information. Individual requests for data access can be directed to the research project leader: jens.christoffer.skogen@fhi.no.
